# An Affecting Letter

**Published:** 1866-08

**Authors:** 


### AN AFFECTING LETTER.

"December 1.

"DEAR SIR:—Will you be so generous as to send a specimen number of THE JOURNAL to one who, besides having been, for the last five years, the unfortunate victim of chronic rheumatism, has recently suffered with evident premonitions of a pulmonary character. I have long desired to avail myself of the valuable information furnished by THE JOURNAL, and indigence (being perpetually dependent upon the charity of my friends) has alone debarred me from that privilege. With a specimen number, I think I shall be able to extend its circulation among the host of visitors (physicians included) who are constantly drawn to my bedside by a desire to witness my (perhaps) unparalleled condition. For several years, I have been stretched perfectly helpless upon my couch, every joint within me as rigid as though I were a mass of stone. My digestive organs are, however, unimpaired, and my intellectual faculties as vigorous as ever; and I am thus enabled, with the aid of a young sister as amanuensis, to instruct a small class of pupils, and to examine such medical works as I am enabled to procure, with a hope of gaining some information which may benefit my health.

"Your reputation for philanthropy encourages me to make this request. Very truly yours."

Chronic Rheumatism, as above, is always the result of the too sudden or long-continued cooling of the body, the fruit of ignorance or foolhardiness. While we admire the philosophical and uncomplaining spirit of our correspondent, and while we extend to him our high respect and warmest sympathies, and while we earnestly call upon the many who have a so much happier lot, because more healthful, to be duly grateful for that happier lot, we cannot but raise a high note of warning to all who can profit by it: TAKE CARE OF YOUR HEALTH WHILE YOU ARE YOUNG! And further, to all parents who have any solicitude for the happiness of their offspring, when they themselves have passed away—*Compel your children to take care of their health!*

